# Ubiquitin-specific protease 11 functions as a tumor suppressor by modulating Mgl-1 protein to regulate cancer cell growth

**DOI:** 10.18632/oncotarget.7581

**Published:** 2016-02-22

**Authors:** Key-Hwan Lim, Bharathi Suresh, Jung-Hyun Park, Young-Soo Kim, Suresh Ramakrishna, Kwang-Hyun Baek

**Affiliations:** ^1^ Department of Biomedical Science, CHA University, Gyeonggi-Do 463–400, Republic of Korea

**Keywords:** deubiquitinating enzyme, RanBPM, UAF1, ubiquitin, USP11

## Abstract

The *Lethal giant larvae (Lgl)* gene encodes a cortical cytoskeleton protein, Lgl, and is involved in maintaining cell polarity and epithelial integrity. Previously, we observed that Mgl-1, a mammalian homologue of the *Drosophila* tumor suppressor protein Lgl, is subjected to degradation via ubiquitin-proteasome pathway, and scaffolding protein RanBPM prevents the turnover of the Mgl-1 protein. Consequently, overexpression of RanBPM enhances Mgl-1-mediated cell proliferation and migration. Here, we analyzed the ability of ubiquitin-specific protease 11 (USP11) as a novel regulator of Mgl-1 and it requires RanBPM to regulate proteasomal degradation of Mgl-1. USP11 showed deubiquitinating activity and stabilized Mgl-1 protein. However, USP11-mediated Mgl-1 stabilization was inhibited in RanBPM-knockdown cells. Furthermore, in the cancer cell migration, the regulation of Mgl-1 by USP11 required RanBPM expression. In addition, an *in vivo* study revealed that depletion of USP11 leads to tumor formation. Taken together, the results indicated that USP11 functions as a tumor suppressor through the regulation of Mgl-1 protein degradation via RanBPM.

## INTRODUCTION

*Lethal giant larvae (Lgl)* is an apical-basal polarity *Drosophila* gene, which functions as a tumor suppressor, controlling the self-renewal and differentiation of progenitor cells. *Lgl* plays a critical role in basal crescent formation [1, 2]. Lgl-1 depleted neural progenitor cells shows loss of cell polarity and asymmetric cell divisions which form neuroblastic rosette-like structures resembling primitive neuroectodermal tumors [3]. A direct interaction between apical proteins is required for basal crescent formation. Lgl-1 provides a functional link between polarity complexes, and this link is essential for cell polarization and asymmetric cell division [4]. As shown by a genomic analysis, *Lgl-1* encodes for a 127 kDa protein with several WD40 repeats predicted to fold into a β-propeller domain involved in protein-protein interactions [5]. Phosphorylation of Lgl-1 by aPKC is also essential for Lgl-1 to perform its different functions. For example, PKC phosphorylates Lgl-1 at the apical cortex of the cell, causing Lgl to disassociate from the cytoskeleton. Lgl-1 remains nonphosphorylated and basally localized in the cortical cytoskeleton, where it anchors for cell fate determinants [6].

Lgl acts as a tumor suppressor. Loss-of-function mutations in *Lgl-1* show neoplastic overgrowth of larval imaginal discs and brain lobes, leading to death at the larval stage in *Drosophila* [7]. The imaginal discs and brain lobes of *Dlg*, *Scrib*, and *Lgl* mutant animals are overgrown and unstructured, and the cells show loss of apical–basal polarity, changing from a columnar to a rounded shape [7–10]. Similarly, Hugl-1, a human homologue of Lgl-1, is down-regulated or completely absent in wide variety of human epithelial malignancies such as breast, lung, prostate, and ovarian cancer and melanomas [11, 12]. Hugl-1 has also been implicated in colorectal cancer progression [13]. Cell adhesion and migration in ovarian carcinomas are associated with gradual cytoplasmic release of Hugl-1 with aPKC basolateral spreading [14].

Recently, we demonstrated that Mgl-1, a mouse homologue of Lgl-1, has tumor suppression activity such as reducing cell proliferation and inhibiting cell migration in Madin Darby canine kidney (MDCK) cells [15]. Mgl-1 functioning might be regulated at multiple levels. At post-translational level, its function is modulated by phosphorylation and ubiquitination [2, 15]. RanBPM, as a scaffolding protein, functionally interacts with and stabilizes Mgl-1 [15]. However, the connection between the stabilization of Mgl-1 by RanBPM and the mechanism of tumor cell suppression is not fully understood.

Ubiquitination and deubiquitination are types of post-translational modifications, and they mainly control the destiny of proteins through 26S proteasomal degradation pathway [16, 17]. Deubiquitinating (DUB) enzymes participate in protein deubiquitination, and they can be classified into at least six subfamilies; ubiquitin-specific proteases (USPs), ubiquitin C-terminal hydrolases (UCHs), Machado–Joseph disease protein domain proteases (MJDs), ovarian tumor proteases (OTUs), JAMM (Jab1/Pab1/MPN metallo-enzyme) motif proteases, and monocyte chemotactic protein-induced proteases (MCPIPs) [18]. USPs comprise the largest subfamily and contain up to 50% of DUB enzymes [19]. Based on crystal structure analysis, most USPs have a USP architecture composed of a palm, thumb, and fingers [20]. The catalytic site of USPs is mostly located in the palm and/or the thumb domains, and the finger domain is responsible for interactions with distal ubiquitin [20]. For example, capturing of ubiquitin by the finger domain of USPs hydrolyzes ubiquitin-ubiquitin or ubiquitin-protein isopeptide bond. USP11 is a DUB enzyme that belongs to the USP family. The biological functions and cellular mechanisms of USP11 are unknown. To gain a better insight into the mechanisms underlying RanBPM-mediated Mgl-1 stabilization, we investigated the stabilization action of USP11 on Mgl-1 in the presence or absence of RanBPM in this study.

## RESULTS

### Mgl-1 interacts with USP11

**Figure 1:**
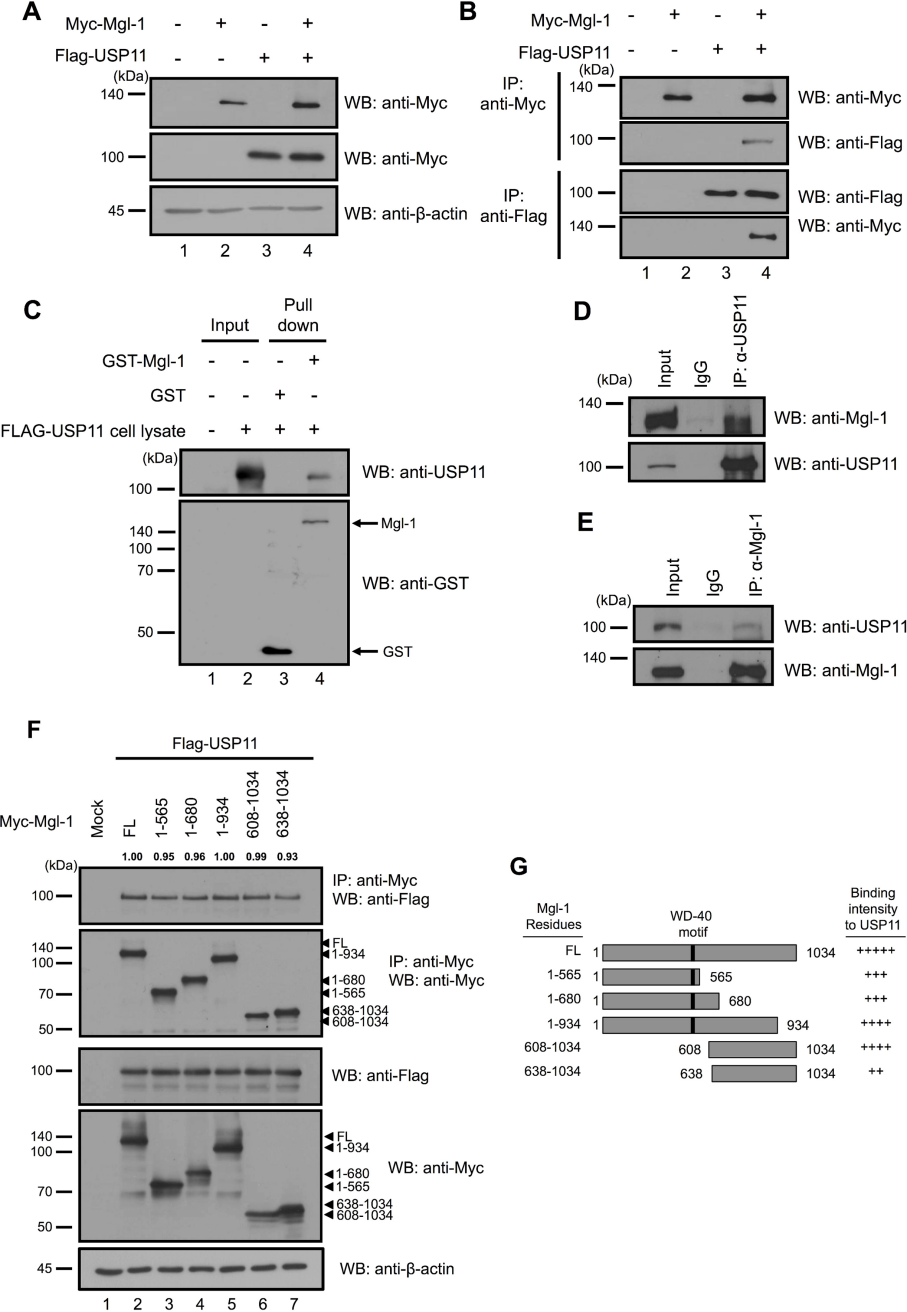
*In vivo and in vitro* interaction between Mgl-1 and USP11 (**A** and **B**) Myc-tagged *Mgl-1* and Flag-tagged *USP11* were co-transfected into HEK293T cells, and immunoprecipitation was performed by using either an anti-Myc or an anti-Flag antibody. Western blotting analysis was performed with indicated antibodies, and β-actin was used as a loading control. (**C**) Recombinant GST or GST-Mgl-1 fusion proteins purified by Glutathione Sepharose^™^ 4B were incubated with cell lysates derived from cells with overexpression of Flag-*USP11*, and the bound proteins were analyzed by immunoblotting with an anti-USP11 antibody. (**D**) MDCK cell lysates were precipitated by an anti-Mgl-1antibody. USP11 and Mgl-1 were detected by appropriate antibodies. (**E**) MDCK cell lysates were precipitated by an anti-Mgl-1 antibody. USP11 and Mgl-1 were detected by appropriate antibodies. (**F**) Myc-tagged *Mgl-1* and its five deletion mutants were co-transfected with Flag-tagged *USP11* into 293T cells. Anti-Myc immunoprecipitation from extract of 293T cells was subjected to immunoblotting with indicated antibodies, and β-actin was used as a loading control. The numbers above the IP blots indicate the relative values from the densitometric quantification. Values are determined as fold changes compared to wild-type Mgl-1. (**G**) Schematic representation of Mgl-1 deletion constructs. All immunoprecipitation bands were adjusted by Image J program and measuring the level of each bands were normalized with immuno-purified Mgl-1. All data performed three independent experiments.

RanBPM interacts with the N-terminal domain of Mgl-1, and the N-terminal domain of RanBPM also interacts with Mgl-1, and these interactions lead to the stabilization of Mgl-1 protein by preventing Mgl-1 degradation [15]. We thought that RanBPM is a scaffolding protein, and it might recruit proteins that inhibit ubiquitination and regulate the turnover of Mgl-1. To gain a better insight into the cellular mechanisms underlying RanBPM-mediated Mgl-1 protein stabilization, we investigated DUB enzymes involved in Mgl-1 ubiquitination. An earlier study reported that RanBPM is a substrate for the DUB enzyme USP11 [21]. To determine whether USP11 directly interacts and regulates Mgl-1 ubiquitination, we co-transfected Myc-tagged *Mgl-1* and Flag-tagged *USP11* into HEK293T cells and subjected them to a co-immunoprecipitation assay using either an anti-Myc or an anti-Flag antibody. To confirm the interaction, we performed reciprocal co-immunoprecipitation of Mgl-1 and USP11 (Figure 1A and 1B). The data showed that Mgl-1 and USP11 readily bind to each other. To reconfirm the interaction between Mgl-1 and USP11, we investigated the interaction between Mgl-1 and USP11 in a Glutathione *S*-transferase (GST) pull-down *in vitro* assay. We performed bacterial recombinant expression of GST-Mgl-1 and immobilized it on a Glutathione-Sepharose matrix. Binding between Mgl-1 and USP11 was established with the GST-pull down assay, and the GST-Mgl-1 fusion protein was incubated with the USP11-transfected cell lysate. GST alone was used as a control sample in this experiment. The USP11 protein was retained by GST-Mgl-1 but not by GST alone (Figure 1C), indicating that Mgl-1 binds to USP11. We next evaluated the endogenous interaction between Mgl-1 and USP11 (Figure 1D and 1E). Mgl-1 was detected by immunoprecipitation using an anti-USP11 antibody, and the reciprocal immunoprecipitation with an anti-Mgl-1 antibody also brought down USP11 (Figure 1D and 1E). The results demonstrate both *in vitro* and *in vivo* interactions between Mgl-1 and USP11. To investigate the protein interaction domain of Mgl-1, we constructed five fragments of Myc-tagged Mgl-1 (1–565, 1–680, 1–934, 608–1034, 638–1034). Using co-immunoprecipitation, we demonstrated that the 1–934 and 608–1034 fragments bind more strongly to USP11 (Figure 1F and 1G). Using densitometric analysis, density of IP blots was quantified, showing similar values compared to the wild-type control (Figure 1F).

These data indicate that USP11 interacts with Mgl-1, and Mgl-1 has multiple binding sites to USP11.

### USP11 deubiquitinates and stabilizes Mgl-1 protein

**Figure 2:**
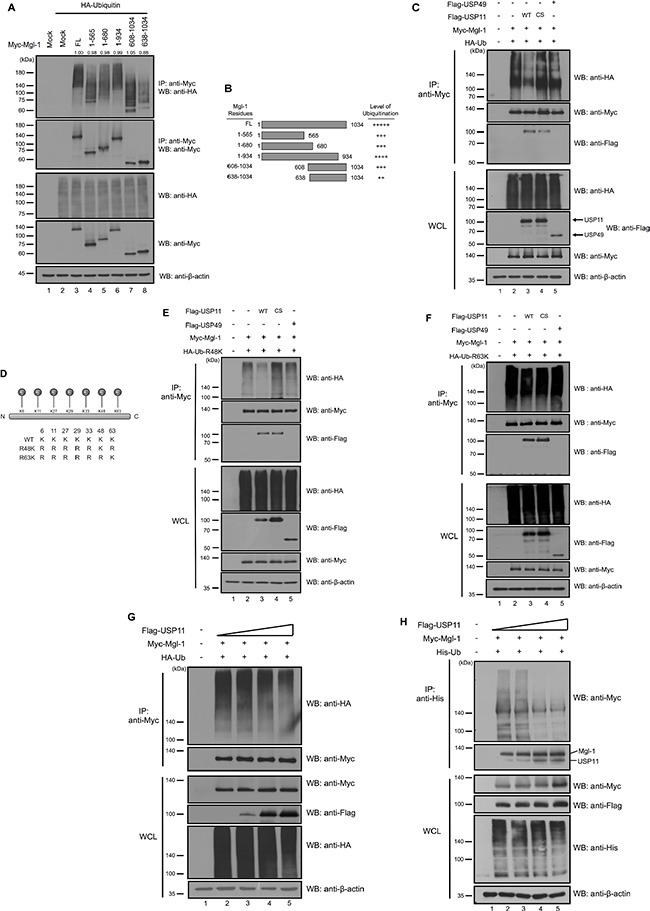
USP11 deubiquitinates Mgl-1 (**A**) 293T cells were transfected with Mgl-1 and its deletion mutants with ubiquitin. The ubiquitination assay was performed by co-immunoprecipitation with an anti-Myc antibody and Western blot using anti-HA and anti-Myc antibodies. The numbers above the IP blots indicate the relative values from the densitometric analysis. Values are determined as fold changes compared to wild-type Mgl-1. (**B**) Schematic representation of Mgl-1 deletion mutants. Smearing bands were adjusted by Image J program and measuring the level of each bands were normalized with immuno-purified Mgl-1. The level of ubiquitination for Mgl-1obtained from three independent experiments. (**C**, **E** and **F**) USP11 has deubiquitinating activity for Mgl-1. 293T cells were transfected with Myc-tagged *Mgl-1*, HA-tagged *ubiquitin* (wild-type, R48K and R63K), Flag-tagged *USP11*, *USP11* (C266S), and *USP49*. Anti-Myc immunoprecipitation from the extract of 293T cells was subjected to immunoblotting with indicated antibodies. (**D**) Schematic representation of ubiquitin lysine mutant constructs. (**G**) USP11 decreased the ubiquitination level of Mgl-1. 293T cells were co-transfected with Myc-tagged *Mgl-1*, HA-tagged *ubiquitin* and increasing amount of Flag-*USP11* (0.5 μg). Immunoprecipitates from 293T cells was subjected to immunoblotting with indicated antibodies. (**H**) Cell lysates from 293T cells transfected with Myc-tagged *Mgl-1*, His-tagged *ubiquitin* and Flag-tagged *USP11* in a dose-dependent manner were subjected to ubiquitination assay with Ni-NTA beads. 2.5 μM of MG132 as a proteasome inhibitor was treated for 4 hrs before cell harvest, and immunoblotting was performed with indicated antibodies.

**Figure 3:**
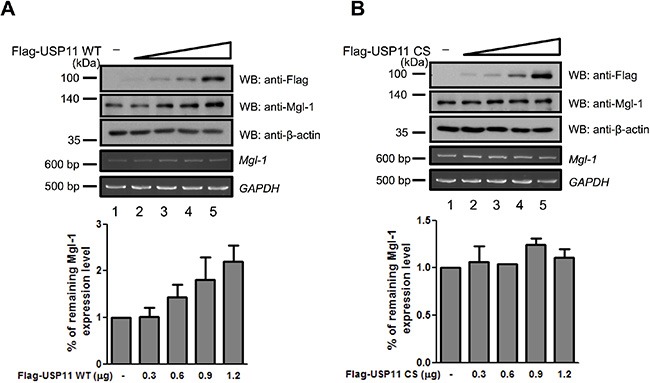
USP11 increases the stability of Mgl-1 by inhibiting protein degradation (**A** and **B**) USP11 increased the expression level of Mgl-1. MDCK cells were transfected with increasing the amounts of wild-type or a catalytic mutant Flag-*USP11* (0.3, 0.6, 0.9 and 1.2 μg), respectively. At 48 hrs after transfection, cell lysates were prepared and subjected to immunoblotting with the indicated antibodies. The expression level of *Mgl-1* gene was analyzed by RT-PCR, and *GAPDH* was used as a loading control. The statistical data were a representative of three biological replicates (*n* = 3, *p < 0.05).

We previously reported that Mgl-1 undergoes polyubiquitination *in vivo* [15]. To determine the ubiquitination domain of Mgl-1, we investigated the ubiquitination level of five Mgl-1 fragments. Five Mgl-1 fragments showed different ubiquitinated Mgl-1 (PolyUb-Mgl-1) size by their molecular weights, and the 608–1034 and 638–1034 fragments showed weaker ubiquitination level compared to the other fragments. Using densitometric analysis, density of IP blots was quantified showing similar values compared to a wild-type control (Figure 2A). It seems that the ubiquitination sites of Mgl-1 are located toward its N-terminal region (Figure 2A and 2B). We next investigated the functional consequence of interaction between Mgl-1 and USP11. We assumed that USP11 may play as a DUB enzyme for Mgl-1 protein. We investigated whether USP11 exhibits deubiquitinating activity on Mgl-1 polyubiquitination. Myc-tagged *Mgl-1* expressing cells were co-transfected with HA-tagged *ubiquitin*, Flag-tagged *USP11*, *USP11* catalytic mutant (C266S) and *USP49* followed by immunoprecipitation with Mgl-1. Interestingly, the expression of USP11 significantly reduced the level of polyubiquitination on Mgl-1 (Figure 2C, lane 3). In contrast, a catalytic mutant USP11 (C266S) and USP49 which were used as negative controls, failed to reduce the ubiquitination level of Mgl-1 (Figure 2C, lanes 4 and 5). It has been reported that ubiquitins are assembled through its Lysine 48 (Lys48) and Lysine 63 (Lys63) residues, which are involved in the 26S proteasome-mediated degradation (Lys48) and distinct signaling pathways (Lys63) [22]. Previously, we have generated ubiquitin mutants that Lys was substituted with Arg (Figure 2D) [15]. Upon overexpression of ubiquitin R48K chains with Mgl-1, we could detect USP11 deubiquitinating activity for Mgl-1 (Figure 2E). However, USP11 did not show deubiquitinating activity for R63K ubiquitin chains on Mgl-1 (Figure 2F). These results indicate that USP11 may prevent Mgl-1 protein degradation from the 26S proteasome. We next gradually expressed USP11 in cells to investigate Mgl-1 ubiquitination. The result showed that dose-dependent expression of USP11 gradually decreased the level of Mgl-1 ubiquitination (Figure 2G and 2H). It is shown that the deubiquitinating activity of USP11 resulted in Mgl-1 protein stabilization, and Mgl-1 protein turnover was regulated by USP11. To elucidate previous results, we confirmed USP11 deubiquitinating activity on Mgl-1 protein stability. We gradually increased the expression with the amount of Flag-tagged wild-type and catalytic mutant USP11, respectively. The level of Mgl-1 protein dramatically was increased with the expression of wild-type USP11 in a dose-dependent manner (Figure 3A). However, the protein level of Mgl-1 was not changed by the expression of catalytic mutant USP11 (Figure 3B). In addition, both wild-type USP11 and a catalytic mutant of USP11 did not affect the mRNA level of *Mgl-1* (Figure 3A and 3B). Our results suggest that USP11 acts as a specific DUB enzyme for Mgl-1 regulating its protein turnover.

### USP11 requires RanBPM to regulate Mgl-1 protein degradation

**Figure 4:**
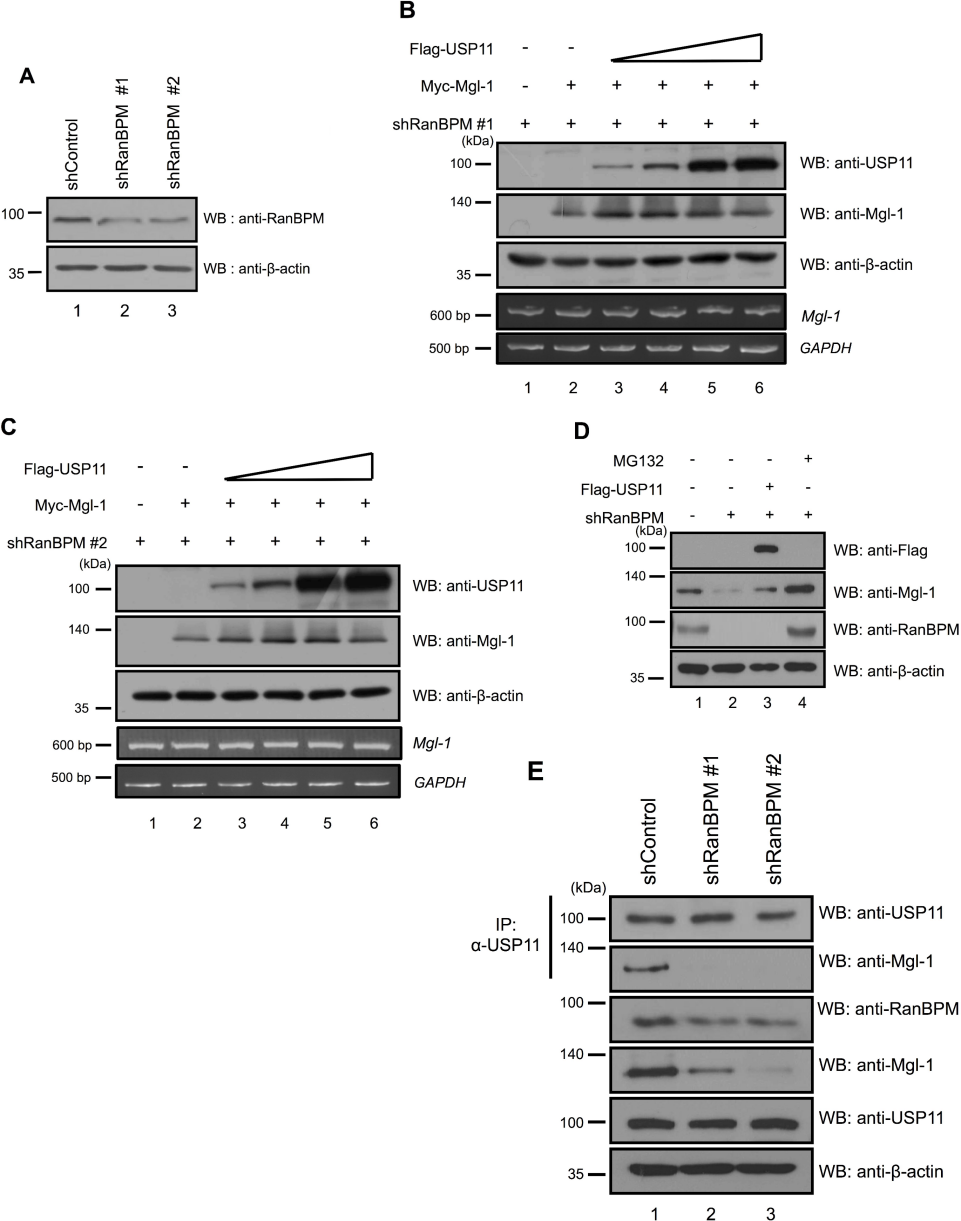
RanBPM recruited USP11 in Mgl-1 protein stabilization (**A**) *RanBPM* shRNAs #1 and #2 were transfected into HEK293T cells and the knockdown effect by each shRNA was analyzed by immunoblotting using indicated antibodies. (**B** and **C**) Immunoblotting assays were performed to determine Mgl-1, USP11, and actin protein levels in stably transfected *RanBPM* shRNA#1 and #2 transfected into HEK293T cells. (**D**) MDCK cells were transfected with *RanBPM* shRNA and Flag-*USP11*, treated with MG132 as a proteasome inhibitor before cell harvest for 8 hrs, and immunoblotted with indicated antibodies. (**E**) Immunoprecipitation with an anti-USP11 antibody followed by immunoblotting with indicated antibodies in *RanBPM* shRNA #1 and #2 transfected into MDCK cells.

The experiments described thus far demonstrate that both RanBPM and USP11 mediate Mgl-1 protein degradation. RanBPM has been known to be associated with several proteins and acts as a protein stabilizer by preventing the degradation of the proteins with which it interacts [15, 23]. Previously, we have reported that RanBPM elongates the half-life of Mgl-1 through the inhibition of ubiquitination for Mgl-1 and inhibition of RanBPM expression leads to decreased level of Mgl-1 [15]. In addition, it interacts with USP11 which is mainly involved in preventing protein degradation of its substrates [24]. Therefore, we hypothesized that RanBPM acts as a mediator for USP11-induced proteasomal degradation of Mgl-1. To test this hypothesis, we investigated the effect of USP11 on Mgl-1 protein stabilization in the absence of RanBPM expression (Figure 4A). Interestingly, the expression of USP11 increased the stability of Mgl-1 protein to some extent (Figure 4B and 4C). Endogenous Mgl-1 level was decreased by knockdown of RanBPM (Figure 4D, lane 2); however, overexpression of USP11 promotes the level of Mgl-1 as previously observed (Figure 4D, lane 3). In addition, both Mgl-1 and RanBPM expression were rescued by MG132 as a proteasome inhibitor treatment in RanBPM-targeting shRNA transfected cells (Figure 4D). To address whether RanBPM can mediate interaction between USP11 and Mgl-1, we used RanBPM knockdown cells. Immunoprecipitation assay was performed with an anti-USP11 antibody and the result showed that interaction between USP11 and Mgl-1 was decreased in knockdown of RanBPM (Figure 4E). This result indicates that RanBPM mediates the interaction between USP11 and Mgl-1. Collectively, these results indicate that USP11 requires RanBPM to regulate Mgl-1.

### USP11 acts as a tumor suppressor by regulating Mgl-1 via RanBPM in cancer cells

**Figure 5:**
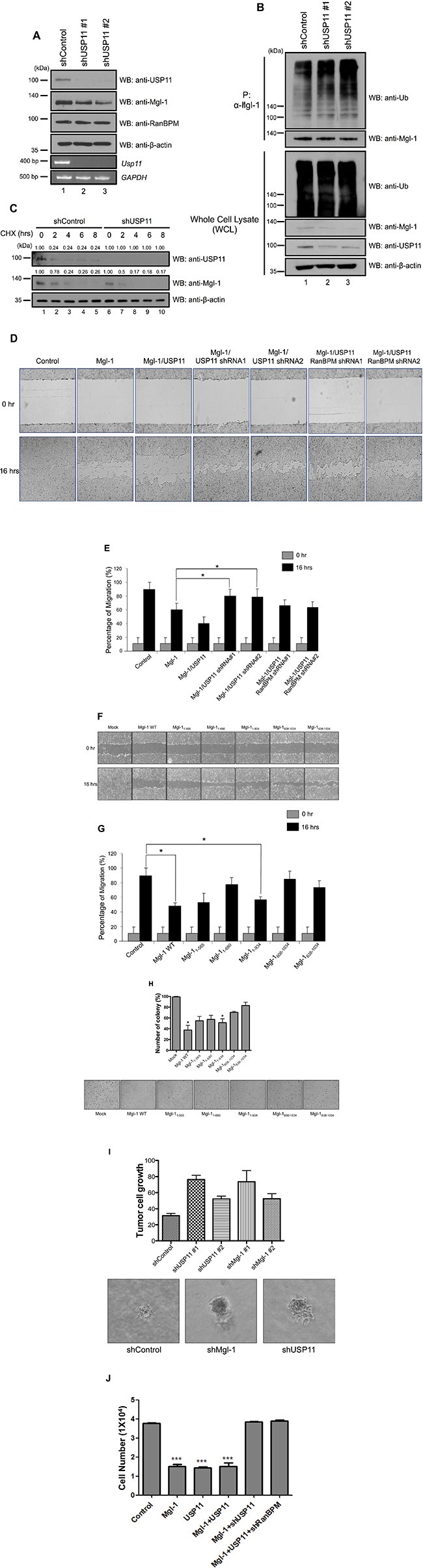
RanBPM regulates Mgl-1-mediated cell migration by recruiting USP11 (**A**) The knockdown efficiency of *USP11* shRNA#1 and *USP11* shRNA#2 was checked by western blot analysis in MDCK cells (upper panel) and its effect on endogenous expression of Mgl-1 (middle panel). RT-PCR was performed for total mRNA from *USP11* shRNA#1 and *USP11* shRNA#2 transfected MDCK cells (lower panel). (**B**) Immunoprecipitation with an anti-Mgl-1 antibody followed by immunoblotting with an anti-ubiquitin antibody showed the smear bands, indicating ubiquitinated Mgl-1. The cell lysates were obtained from the *USP11* shRNA #1 and #2 transfected A549 cells and immunoblotting was performed with indicated antibodies. (**C**) Depletion of USP11 reduces the half-life of Mgl-1. A549 cells were transfected with scrambled or *USP11* shRNA. After 48 hrs of transfection, cells were treated with cycloheximide and harvested at the indicated time. Cell extracts were used for immunoblotting with indicated antibodies. The numbers above each blot indicate the relative values from the densitometric analysis. Values are determined as fold changes compared to CHX-untreated cells. (**D** and **F**) Wound healing assays were used to determine the Mgl-1-mediated migratory and invasive potential of stably transfected MDCK cells with respective constructs. Images were captured at a time interval between 0 and 16 hrs. Assays were performed in triplicate. (**E** and **G**) The percentage of migration was statistically analyzed in three separate experiments. *n* = 3, *p < 0.05. (**H**) A549 cells were transfected with indicated constructs. After transfection, the cells were plated in soft agar that included with G418 to select transfected cells, and transformed cells were counted 3 weeks later. *n* = 3, ***p < 0.005. (**I**) A549 cells were infected with lenti-virus expressing *USP11* shRNA or a control vector. After infection, the infected cells were selected with puromycin. The selected A549 cells were plated in soft agar, and transformed cells were counted 2 weeks later. *n* = 3, *p < 0.05. (**J**) Cell counting was performed with the cells transfected with indicated genes.

We previously showed that the interaction between RanBPM and Mgl-1 enhanced the tumor-suppressing activity of Mgl-1 by reducing cell migratory ability in mammalian cell lines [15]. Based on our previous observation demonstrating the role of USP11 in RanBPM-mediated Mgl-1 protein degradation, we hypothesized that USP11 might regulate Mgl-1 cell migration via RanBPM. We investigated the effect of USP11 on Mgl-1-mediated cell migration in RanBPM-deficient cells. We generated USP11 depleted cells with two shRNAs against *USP11* mRNA that have different target mRNA sequence on *USP11* (Figure 5A). As expected, the depletion of USP11 decreased Mgl-1 expression and increased ubiquitination level of Mgl-1, but the mRNA level of *Mgl-1* was decreased by the depletion of USP11 (Figure 5A and 5B). We next determined the half-life of Mgl-1 in the absence of USP11. Cells transfected with shRNA against *USP11* were treated with cycloheximide (CHX), and we found that Mgl-1 was shown to have a half-life of less than 6 hrs in control cells, whereas the degradation of Mgl-1 in USP11 depleted cells was faster than that of the control (Figure 5C). Density of each blot was quantified by densitometric analysis. Values of blots against USP11 and Mgl-1 were decreased in a time-dependent manner after CHX treatment (Figure 5C). We also analyzed the migratory behavior of the USP11 shRNA transfected MDCK cells, and the result indicated that the Mgl-1-transfected cells showed significantly reduced migration when compared to the controls (Figure 5D). Co-transfected *Mgl-1* and *USP11* cells significantly reduced migration in comparison with cells transfected with Mgl-1 alone (Figure 5D and 5E). Interestingly, RanBPM knockdown by RanBPM shRNA prevented USP11-induced Mgl-1 cell migration in the MDCK cells (Figure 5D and 5E). These results indicate that USP11 regulates cell migration through an association with Mgl-1 and that RanBPM acts as a mediator for USP11. Previously, we identified the interaction domain of Mgl-1 with five fragments of Myc-tagged Mgl-1 (1–565, 1–680, 1–934, 608–1034, 638–1034), and 1–934 and 608–1034 fragments strongly interacted with USP11 (Figure 1F and 1G). We tested whether these Mgl-1 fragments can affect cell migration and the number of colony formation. The results showed that 1–934 fragment which binds USP11 the most strongly reduced cell migration (Figure 5F and 5G), and inhibit colony formation (Figure 5H). Other fragments (1–565 and 1–680) also reduce cell migration and colony formation (Figure 5F and 5H). Because threeMgl-1 fragments (1–565, 1–680, 1–934) have the WD-40 motif (Figure 1G) and they are associated with USP11 (Figure 1F), it is suggested that three Mgl-1 fragments might have effect on cell proliferation as shown with wild-type Mgl-1. We next examined the formation of cancer cell colony on soft agar to investigate the loss of USP11 function *in vivo*. A549 lung adenocarcinoma cells that stably inhibit USP11 increased colony formation (Figure 5I). Finally, we counted live cells which induced the expression of Mgl-1 and/or USP11 in the absence of USP11 and RanBPM, respectively. Overexpression of Mgl-1 did not affect the cell proliferation in both USP11 and RanBPM knockdown condition (Figure 5J). Taken together, these findings indicate that RanBPM interacts and cooperates with USP11 to stabilize and enhance Mgl-1-mediated tumor suppression in mammalian cell lines.

### USP11 suppresses Mgl-1 mediated cell polarity

**Figure 6:**
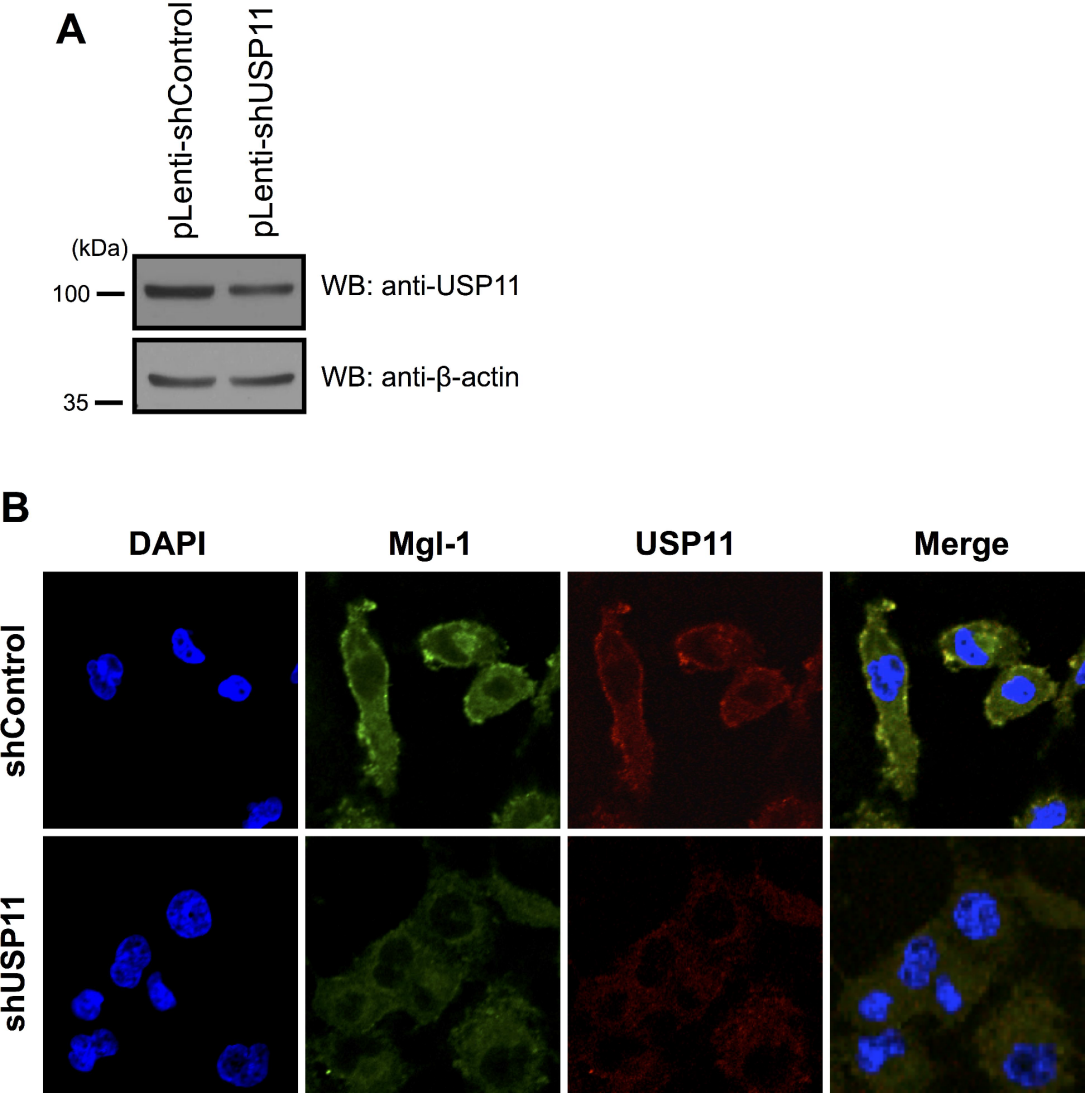
Knock down effect of USP11 on cancer cells (**A**) Knockdown effect on A549 cells that were infected with *USP11* shRNA or a control vector-encoded lenti-virus. (**B**) USP11 regulated Mgl-1 polarity. A549 cells were infected with lenti-virus expressing *USP11* shRNA or a control vector. Lenti-virus-infected cells were stained with anti-Mgl-1 (green), anti-USP11 (red) and DNA was stained with DAPI.

Mgl-1 is localized in the cell membrane where it regulates cell polarity as previously described [14]. Previously, we confirmed that depletion of USP11 decreases the expression level of Mgl-1 (Figure 5A) and increases the tumor cell growth (Figure 5F). In addition, presence of Mgl-1 reduced cancer cell proliferation (Figure 5D–5H) [15]. We hypothesized that USP11 may play a role in maintaining cell polarity through regulation of half-life of Mgl-1. We next investigated whether USP11 involved in the cell polarity through regulation of Mgl-1 in cancer cells. We established a stable cell line with depleted USP11 using a lenti-viral system and the cells were analyzed by immunofluorescence analysis (Figure 6). Specific antibodies were used in pairwise combinations to directly compare the respective expression patterns of these proteins. Interestingly, the expression level of Mgl-1 in cell membrane was not observed in the cell membrane in the absence of USP11 compared to control cells as we expected (Figure 6A and 6B). Collectively, this result indicates that USP11 regulates the localization of Mgl-1.

## DISCUSSION

In this study, we demonstrated a direct interaction between USP11 and Mgl-1. With the presence of RanBPM, USP11 deubiquitinates Mgl-1 to stabilize, indicating that RanBPM is essential for Mgl-1 stabilization. USP11 suppresses tumor cell growth via the regulation of Mgl-1, and the suppression requires RanBPM expression. Furthermore, inhibition of USP11 leads to the suppression of Mgl-1 polarity to the cell membrane. Taken together, the results indicate that USP11 functions in cancer cells and that it regulates Mgl-1 via RanBPM, thereby serving as a platform for the regulation of the function of Mgl-1 through ubiquitination and deubiquitination.

Ubiquitination is a post-translational modification, in early embryonic development [29]. To gain better insight into the mechanisms underlying Mgl-1 tumor suppression, we aimed to identify cellular interactions of Mgl-1 involved in the regulation of its tumor-suppressor activity. We identified a scaffolding protein RanBPM. RanBPM was previously characterized as a binding protein of Ran, a small GTPase and as a novel binding partner of Mgl-1 by yeast two-hybrid screening [15].

RanBPM shows diverse interactions, complexes in different cellular environments, and regulates various cellular processes [30] RanBPM acts as a protein modulator [15, 23], transcriptional regulator [31–33], and cell cycle regulator [34, 35]. Recently, we showed that RanBPM interacts and modulates the stability and biological functions of Mgl-1 [15]. A lethal giant larvae (Lgl), a part of the cytoskeletal protein complex, is involved in establishing polarized epithelia, cell proliferation, cell differentiation and tissue organization [8, 36]. RanBPM inhibits Mgl-1-mediated cell proliferation and cell migration, suggesting that it modulates the tumor-suppressor activity of Mgl-1 [15]. RanBPM also increases the level of the Mgl-1 protein in a dose-dependent manner and extends its half-life by preventing Mgl-1 protein degradation [15]. RanBPM exhibits a similar type of action in RanBPM-mediated regulation of p73α, an oncogenic protein [23]. The binding of RanBPM to p73 results in nuclear translocation of cytoplasmic RanBPM and stabilization of the p73 protein by preventing its degradation through the ubiquitin proteasomal pathway. As a functional consequence, RanBPM enhances the transcriptional activity of p73α and subsequently increases the proapoptotic function of p73α [23]. Taken together, the function of RanBPM on p73α and Mgl-1 indicates a novel role of RanBPM as a protein stabilizer.

The role of Lgl as a tumor suppressor is well known, with loss-of-function mutations in Lgl resulting in neoplastic overgrowth of larval imaginal discs and brain lobes, leading to the death of *Drosophila* at the larval stage [7]. Similarly, down-regulation of Hugl-1 correlates with the occurrence of colorectal cancers, and its expression leads to an increase in cell adhesion and decrease in cell migration [13]. Hugl-1 plays a key role in the regulation of proteins involved in the epithelial-mesenchymal transition (EMT), a process that enables epithelial cells to gain mesenchymal and migratory properties [12]. Lgl2, a second *Lgl* orthologue, also acts as a tumor suppressor in zebra fish epidermis and is involved in EMT [37]. It is suggested that the Lgl protein primarily acts as a tumor suppressor. In mouse embryonic fibroblasts, a mutant of Mgl-1 lacking five serine residues reduced cell polarization in an *in vitro* wounding assay [38]. Mgl-1 is phosphorylated in the Mgl-1-aPKC-mPar-6 complex by a process that requires five closely spaced serine residues located in PKC consensus phosphorylation sites, suggesting that aPKC is a functional Mgl-1 kinase [38].

Here, we identified USP11 as a binding partner of Mgl-1 and illustrated its role in the regulation of cancer cell proliferation and growth. RanBPM is associated with USP11, which is generally involved in preventing ubiquitination of targeted proteins [21]. Therefore, we speculated that RanBPM promotes the deubiquitination of Mgl-1 by recruiting USP11 to Mgl-1. The research was initiated to identify specific DUB enzymes of Mgl-1 because understanding of the post-translational regulation of Mgl-1 is essential to elucidate the functional molecular mechanism of Mgl-1-mediated tumor suppression. Our present study clearly suggests that the stability and activity of Mgl-1 are regulated by USP11 through a direct protein-protein interaction. USP11 formed a stable complex with Mgl-1 and positively regulated its stability and activity. In contrast, a catalytic mutant of USP11 did not show stabilizing behavior as compared with wild-type USP11. Based on the function of RanBPM as a protein adaptor, we suspected that a complex might be involved in the mutual interaction among Mgl-1, RanBPM, and USP11. Therefore, we examined the stabilizing action of USP11 on Mgl-1 in the presence or absence of RanBPM. Endogenous depletion of RanBPM inhibited the stabilizing action of USP11 on Mgl-1, indicating the crucial role of RanBPM in controlling the deubiquitination of Mgl-1 by USP11. Cells lacking USP11 increased cancer cell migration and growth via RanBPM. Moreover, USP11-depleted cells failed cell polarization. Collectively, our results indicate that USP11 acts as a tumor suppressor through the regulation of Mgl-1 and that it requires RanBPM as a binding partner to deubiquitinate Mgl-1. This finding is similar to the previous observations that the activity of some DUB enzymes requires substrates as a chaperone [39, 40]. For example, UAF1 is required for full catalytic activity of USP1, USP12, and USP46 [40]. USP1 regulates the mono-ubiquitination of FAND2 in the Fanconi anemia (FA) pathway, and it requires UAF1 to have deubiquitinating activity for regulation of Ub-FANCD2 deubiquitination [40–42]. This observation indicates that UAF1 activates the DUB enzyme activity of USP1 and that it functions as a scaffolding protein for USP1 targeting FANCD2 [40, 43].

**Figure 7:**
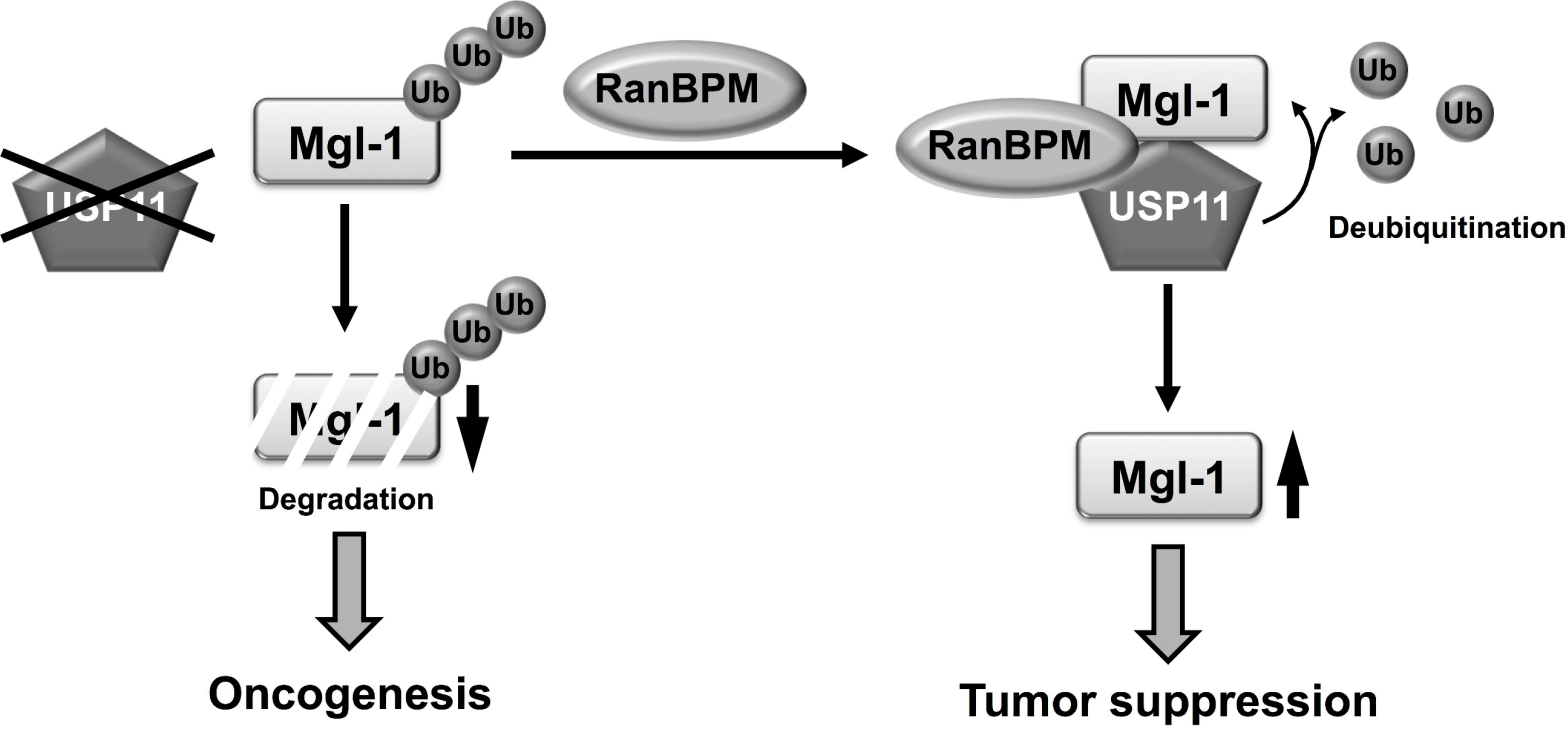
A schematic model of USP11 regulation Dissociation of Mgl-1 from USP11 in the absence of RanBPM reduced the stability of Mgl-1 by ubiquitination. However, RanBPM recruited USP11 to Mgl-1, and USP11 detached ubiquitin from Mgl-1 by its deubiquitinating enzyme activity. Deubiquitination of Mgl-1 by USP11 led to the elongation of Mgl-1 turnover. Finally, their interaction regulated tumorigenesis.

The level of the Mgl-1 protein is important for its diverse functions, but little is known about the post-translational modification of Mgl-1, especially at the level of protein degradation. In the present study, we identified the interaction between USP11 and Mgl-1. The removal of ubiquitin conjugates from target proteins by DUB enzymes has emerged as an important regulatory mechanism in a wide range of cellular processes [44, 45]. Although it has been suggested that approximately 100 functional DUB enzymes are encoded by the human genome, the functions or substrates of most of these have yet to be assigned [46]. Moreover, studies of the tumor-suppressive function of USPs are extremely limited. For example, the p53 known as a tumor suppressor and checkpoint protein for DNA damage is regulated by several USPs, including USP7 (known as HAUSP), USP10, USP29 and USP42 in various tumors [47–50]. USP7 is known as the first DUB enzyme for p53, and it regulates p53 ubiquitination and increases p53 stability via Mdm2 [47]. USP29 is also a specific p53-associated DUB enzyme; however, it requires a co-activator JTV1 to stabilize p53 [49]. These observations indicate that several DUB enzymes have tumor-suppression functions, and that their functions depend on the presence of binding partners. It may be that more than one DUB enzyme interacts with Mgl-1 and regulate its biological functions to suppress tumor growth. This hypothesis motivated the current research. Our results revealed that USP11 is a novel DUB enzyme in cancer cells. Targeting the ubiquitin-proteasome system has been trying to identify cancer specific drugs. However, the side effect in treatment for cancer should be considered. Our findings suggest a new aspect for the cancer therapy as USP11 can be a putative tumor suppressor, but more research is required to clarify the function of USP11 in cancer. Taken together, our findings underscore the importance of USP11 as a new tumor suppressor in the regulation of the Mgl-1 function via RanBPM (Figure 7). To understand the complex functions of Mgl-1, identification of additional substrate-specific DUB enzymes involved in Mgl-1 regulation is necessary.

## MATERIALS AND METHODS

### Cell culture and transfection

HEK293T (human embryonic kidney cell line), MDCK (Madin Darby canine kidney cell line), A549 (Human lung adenocarcinoma epithelial cell line) and HeLa (human cervical cancer cell line) were cultured in DMEM (GIBCO BRL, Rockville, MD, USA) supplemented with 10% fetal bovine serum (GIBCO BRL, Rockville, MD, USA) and 1% penicillin and streptomycin (GIBCO BRL, Rockville, MD, USA). Transfection was carried out using polyethyleneimine (Polysciences, Warrington, PA, USA) and Lipofectamin 2000 (Invitrogen, Carlsbad, CA, USA).

### Construction of expression plasmids and shRNA constructs

The cDNA encoding a full-length *Mgl-1* and deletion forms of *Mgl-1* (1–565 aa, 1–680 aa, 1–934 aa, 608–1034 aa, and 638–1034 aa) were subcloned into pcDNA3.1 expression vector (Invitrogen, Carlsbad, CA, USA), and all ubiquitin constructs were subcloned into pCS4-HA vector as previously described [15]. In order to obtain a full-length cDNA for *USP11*, RT-PCR was performed with reference to cDNA coding sequence of mouse *USP11* in GenBank (NM_145628). RNA isolated from NIH3T3 cells served as templates for reverse transcription. The amplified DNA fragment was gel purified and subcloned into pCS4–3-Flag expression vector and then subjected to sequence analysis. *USP49* cDNA was cloned using mRNA from HeLa cells, and subcloned into pCS4–3-Flag vector. The pCS4-HA and pCS4–3-Flag vectors are kindly provided by Dr. J.H. Kim (CHA University, Korea). To generate an inactive *USP11* mutant, the active site residue cysteine at the position 266 was substituted with serine by site-directed mutagenesis. The full-length *Mgl-1* was subcloned into pGEX4T-1 vector (Pharmacia Biotech, Piscataway, NJ, USA) for GST pull-down assays. Flag-tagged *RanBPM* was kindly provided by Dr. Yoshiaki Ishigatsubo (Yokohama City University School of Medicine, Japan). The shRNA expression vectors for *USP11 and RanBPM* were constructed using the pSilencer 1.0-U6 vector (Ambion, Austin, TX, USA). The insert sequences used for designing shRNA are CAA CTC CTA CTA TGG TCT G for USP11 shRNA#1, TGA GAA TCA GAT TGA GTC C for USP11 shRNA#2, GGA CAA GTT CAG CTA CAT C for RanBPM shRNA#1, and CAA TAC CTG CTT TTA CAC C for RanBPM shRNA#2 (UbProtein Inc., Seongnam, Korea).

### Antibodies

To generate a polyclonal antibody against USP11, its C-terminal region (798–921 amino acids) was cloned into pGEX4T-1 vector (Pharmacia Biotech, Piscataway, NJ, USA) and expressed in BL21 cells. Recombinant GST-tagged protein was purified by a Glutathione Sepharose column (Pharmacia Biotech, Piscataway, NJ, USA). After the final booster injection, the whole blood was drawn and the serum was separated. A mouse anti-9E10 monoclonal antibody, a mouse anti-HA monoclonal antibody, an anti-RanBPM, and an anti-b-actin monoclonal antibody were purchased from Santa Cruz Biotechnology (Santa Cruz Biotechnology, Santa Cruz, CA, USA). An anti-ubiquitin antibody was purchased from Cell Signaling (Cell Signaling, Beverly, MA, USA). Mgl-1 antibody was kindly provided by Patrick J. Brennwald (University of North Carolina at Chapel Hill, USA.

### Pull-down assay

HEK293T cells were transfected with Flag-*USP11*. Cells were harvested after 48 hrs and suspended in buffer A (20 mM Tris-HCl, 1 mM EDTA, 1 mM dithiothreitol, 150 mM NaCl, 1% Triton X-100). The cell extract was incubated with GST-Mgl-1 immobilized on 100 μl of Glutathione-Sepharose beads (Pharamacia Biotech, Uppsala, Sweden). Beads were washed with buffer A. The bound proteins were eluted by boiling in the SDS sample buffer and immunoblotted with an anti-USP11 antibody and an anti-GST antibody.

### *In vivo* co-immunoprecipitation assay

Myc-tagged *Mgl-1* and Flag-tagged *USP11* were transfected individually and co-transfected in HEK293T cells. Cells were lysed after 48 hrs and immunoprecipitated with an anti-Myc, an anti-Flag or an anti-HA antibody and immunoblotted with respective antibodies to check the binding interaction.

### *In vivo* ubiquitination and deubiquitination assays

For ubiquitination of Mgl-1, cells were transfected with Myc-*Mgl-1* and HA-*ubiquitin*. After 48 hrs, cells were harvested and lysed in buffer B (50 mM Tris [pH 7.6], 150 mM NaCl, 1 mM EDTA, 1% Triton X-100, 1 mM PMSF) for 20 min. Cell lysates were incubated with the corresponding antibodies at 4°C overnight and then incubated with 20 μl of protein A/G Sepharose at 4°C for 1 hr. Beads were washed with a lysis buffer and elution was performed with SDS sample buffer, and co-immunoprecipitated proteins were detected by Western blotting. For ubiquitination and deubiquitination assays, cells were transfected with Myc-*Mgl-1*, Flag-*USP11*, Flag-*USP11* (C266S), and HA-*ubiquitin*. Cells were harvested and immunoprecipitation assays were performed as described above.

### Ubiquitination assay with Ni-NTA beads

HEK 293T cells co-transfected with Myc-tagged *Mgl-1*, Flag-tagged *USP11*, and His-tagged *ubiquitin* were lysed in urea lysis buffer (8 M urea, 100 mM NaPO_4_ (pH 8.0), 15 mM imidazole). Cell lysates were mixed with Ni-NTA beads (Novagen, Madison, WI, USA) at room temperature for 4 hrs. After reaction, beads were washed with washing buffer (6 M GuHCl, 0.1% Triton X-100, 100 mM NaPO_4_ (pH 8.0), 15 mM imidazole) two times, and finally washed with urea lysis buffer.

### Soft agar assay

A549 cells were suspended in complete DMEM containing 0.4% agar and seeded in 6-well plates with 0.6% base agar in complete DMEM, and cells were incubated in 37°C humidified incubator. After 2 weeks, colonies were photographed and counted.

### Wound healing and cell counting assays

Stably transfected MDCK cells were cultured with near 90% confluence. A scratch was made on MDCK monolayer with a sterile pipette tip in a definite array. The wounded cell layer was washed with PBS and incubated with complete medium and wound closure was captured at 0 hr and 16 hrs and measured by DMC Advanced 3.1 program. Each set of samples was analyzed in triplicate. For live cell counting, transfected cells were cultured at a density of 1 × 10^4^ cells for 4 days and viable cells were determined by trypan blue exclusion.

### Lenti-viral infection

For lenti-viral infection, shRNA against *USP11* cloned into pLKO-puro lenti-virus encoding vector and A549 cells were infected with pLKO-control and pLKO-*USP11* lent-virus respectively. After infection with the lenti-virus, infected A549 cells were selected with puromycin (3 μg/ml).

### Immunofluorescence

For immunofluorescence assay, stably *USP11* knockdown and control cells by lenti-virus were grown on glass coverslips and fixed in 4% paraformaldehyde in PBS for 10 min at room temperature. After washing in PBS, the cells were permeabilized in PBS / 0.1% Triton for 5 min, washed extensively in PBS and then incubated with primary antibody diluted in PBS for 1 hr followed by the appropriately conjugated secondary antibodies. The cells were then washed several times in PBS and mounted on glass slides. Cells were visualized by using a Zeiss Axiovert 100 M microscope (Carl Zeiss, Oberkochen, Germany) attached to an LSM 510 confocal unit.

### Statistical analysis

We adjusted the bands from all Western blot results by a computer program (Image J) for statistical analysis. Softwares Microsoft Excel and Prism were used for all statistical analysis, and significances of calculated data were expressed as *p*-values. The intensity of specific bands from Western blotting was quantified by densitometric analysis and expressed as fold changes compared to the expression of a control.
